# Case report: Colonic bezoar due to Box Myrtle seeds: A very rare occurrence

**DOI:** 10.4103/0971-3026.76049

**Published:** 2011

**Authors:** Narvir Singh Chauhan, Dinesh Sood

**Affiliations:** Department of Radiodiagnosis, Dr. Rajendra Prasad Government Medical College, Tanda, (Kangra), Himachal Pradesh - 176 001, India

**Keywords:** Box Myrtle, colonic bezoar, hyperechoic arc, kaiphal, seed bezoar, sigmoid stricture

## Abstract

Large bowel seed bezoars are rare and are mostly due to seeds of sunflower, prickly pear cactus, sesame and cucumber. We present a rare case of sigmoid colon seed bezoar due to Box Myrtle seeds because of an underlying benign stricture.

## Introduction

A bezoar is the result of the ingestion of indigestible or poorly digestible substances that accumulate in the gastrointestinal tract in the form of a mass. It can result from any substance that is capable of forming concretions within the gastrointestinal tract. The classification of a bezoar is according to the foreign material that constitutes its core and includes - trichobezoar (hair), phytobezoar (fruits or vegetable fibers), lactobezoar (milk curd), lithobezoar (rock-like substances) or combinations like trichophytobezoars.[1,2] Cases of bezoars due to unusual materials like medications, shellac, cement, cotton, dry fruit and cushion foam, etc. can occur rarely.[2–6] The stomach is the most common site of bezoar formation, followed by the small intestine and, rarely, the colon or rectum.[7,8] Large bowel seed bezoars are uncommon, and most cases reported have been rectal bezoars presenting clinically with fecal impaction.[9,10] We describe a rare case of a colonic seed bezoar due to Box Myrtle seeds.

## Case Report

A 35-year-old female patient presented in the surgery department with dull abdominal pain in the left paraumblical region. She gave a history of progressively worsening obstipation for the past 2 years for which she was using laxatives. No clinical features of obstruction were present. Her routine laboratory tests were normal, except for mild leukocytosis.

**Figure 1:**
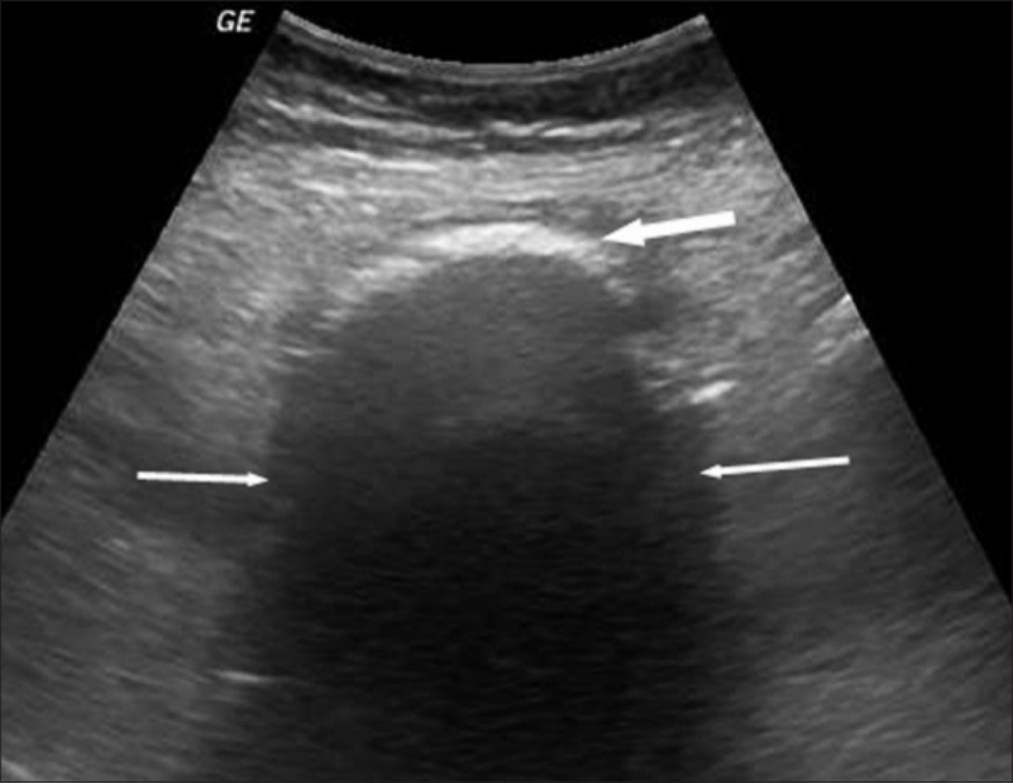
USG of the left lower abdomen showing a hyperechoic arc (thick arrow) with a dense posterior acoustic shadow (thin arrows) in the location of the sigmoid colon

**Figure 2:**
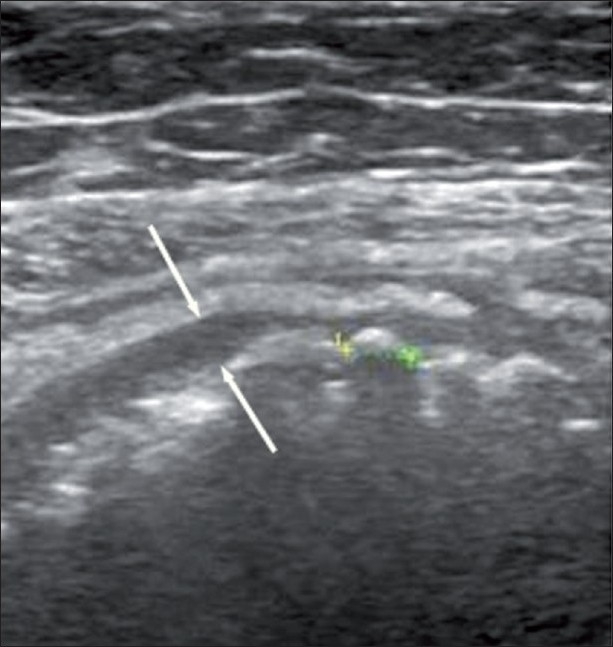
USG with a high-frequency probe showing the individual seeds (between cursors) and mild wall thickening of the sigmoid colon (thin arrows)

**Figure 3:**
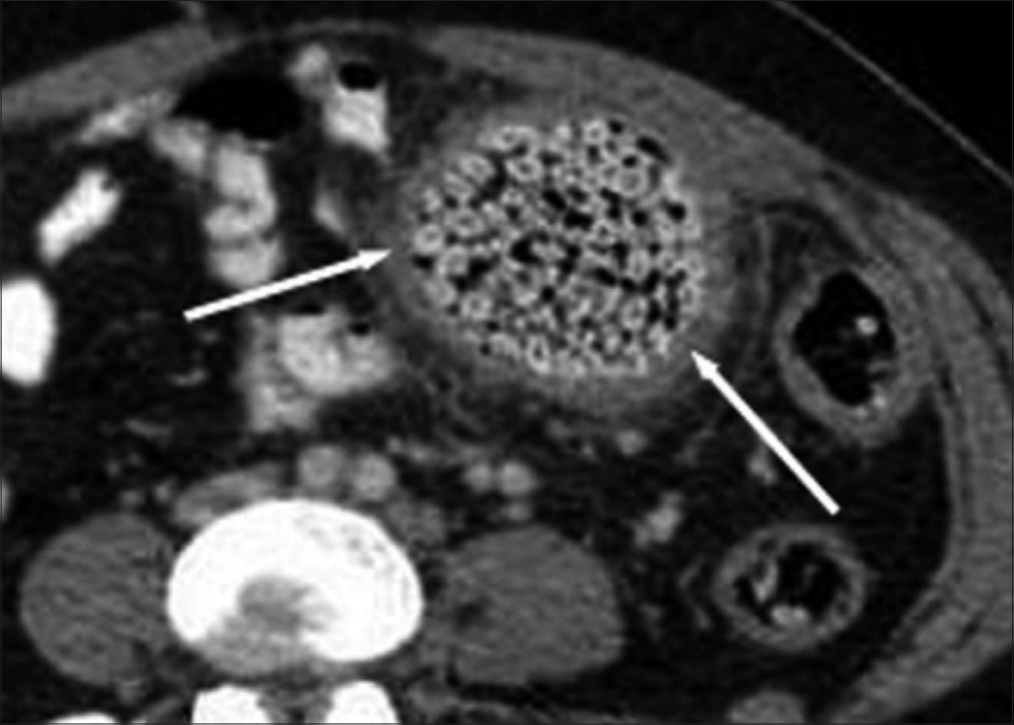
Axial contrast-enhanced CT scan showing the sigmoid colon seed bezoar along with the inflammatory wall thickening (arrows)

**Figure 4:**
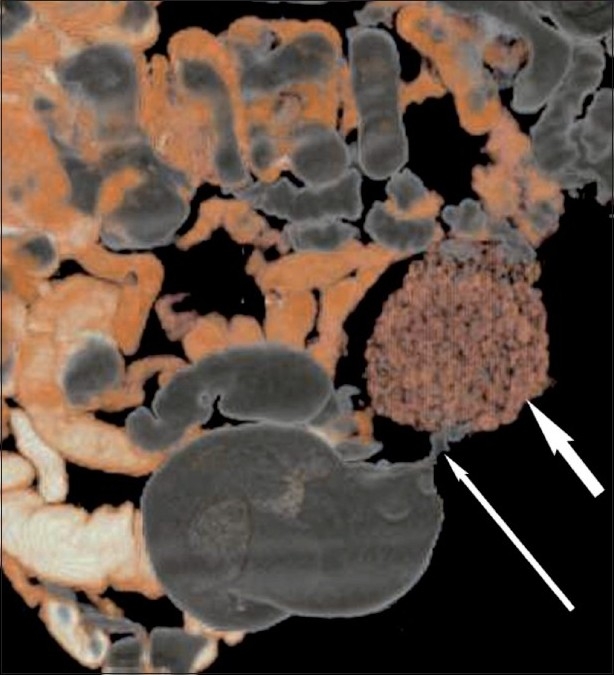
Volume-rendered CT scan showing the colonic seed bezoar (thick arrow) proximal to the sigmoid stricture (thin arrow)

**Figure 5:**
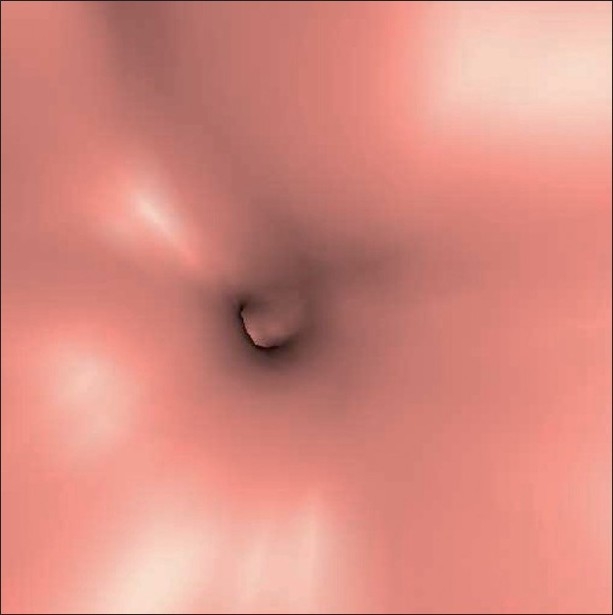
Volume-rendered CT endoscopy image showing a tight sigmoid stricture (viewed from below)

The abdominal radiograph showed localized mottled lucencies in the left lower abdomen. USG revealed mild wall thickening in the region of the sigmoid colon with the presence of an intraluminal, curvilinear, hyperechoic arc-like surface casting a prominent posterior acoustic shadow [Figure 1]. Scanning with a linear high-frequency probe revealed the arc to be composed of multiple, tiny 4–8-mm-sized hyperechoic shadows [Figure 2]. On questioning, the patient recalled having consumed large quantities of the wild fruit *“Kaiphal”* or Box Myrtle along with the seeds about 3 weeks ago. Based on the USG findings and history, a diagnosis of colonic seed bezoar was suggested. Contrast-enhanced CT scan of the abdomen revealed the presence of a mass-like lesion in the sigmoid colon involving an approximately 7-cm segment. It was composed of multiple tightly packed ovoid densities having a seed-like appearance, the size of individual seeds ranging from 4 to 8 mm. These showed a hyperdense periphery (CT attenuation value, 120–140 HU) with a relatively hypodense center (CT attenuation value, 50–70 HU) with air in the interstices, giving a mottled appearance. Mild associated inflammatory changes were present with circumferential mural thickening (3-4 mm) and stranding of the pericolonic fat [Figure 3]. A tight, short segment stricture was seen immediately distal to the colonic bezoar [Figures 4 and 5]. No evidence of a soft tissue mass, regional lymphadenopathy or ischemia was present.

The patient was taken up for colonoscopy and the stricture was dilated. The seed bezoar was disimpacted and 300 g of largely undigested seeds were removed. Biopsy taken from the stricture revealed fibrosis, and no evidence of malignancy was detected.

## Discussion

Seed bezoars occur after consuming large quantities of seeds either by eating the fruit together with the seeds or by eating the seeds themselves. In one retrospective study in Israel on fecal impaction by seed bezoars in the rectum in hospitalized patients, phytobezoars were found in 30 patients. The seed bezoars were composed of prickly pear seeds in 12 patients, watermelon seeds in 10 patients, sunflower seeds in 4 patients and popcorn kernel and pomegranate seeds in one patient each.[10]

*“Kaiphal,”* also known as Box Myrtle or *Myrica esculenta*, is a tree found abundantly in the Indian Himalayas. It bears small berry-like fruits that are usually consumed without seeds. Our patient had consumed large quantities of this fruit along with the seeds because of a lack of awareness. The seeds then became impacted in the sigmoid colon due to the presence of a benign short segment stricture. There is one case report of a patient who presented with large bowel obstruction, which was found to be due to a sesame seed bezoar in the sigmoid colon proximal to a benign postoperative anastomotic site stricture.[11]

While plain radiographs of the abdomen may be useful in the evaluation of intestinal obstruction, bezoars themselves are only rarely identified. This is because the imaging feature of a bezoar is not characteristic, and can be mistaken for feces or abscess. USG may show the presence of an intraluminal mass with a hyperechoic arc-like surface showing marked acoustic shadowing[12] and may also detect any associated intestinal obstruction. Our patient had typical USG findings.

CT scan is considered to be the imaging modality of choice for confirming gastrointestinal bezoars, and shows an intraluminal mass with air in the interstices or an inhomogeneous mass with a mottled gas appearance. CT scan also determines the point of obstruction; it can confirm that the bezoar is the underlying cause of obstruction and also helps detect the existence of additional bezoars.[13] Our patient showed typical findings, and individual seeds could also be identified as they were largely undigested.
